# Provident Dispensaries

**Published:** 1901-07-06

**Authors:** 


### PROVIDENT DISPENSARIES.

At a recent meeting of the Metropolitan Provident Medical Association held by invitation of Mr. W. F. D. Smith, M.P., and Lady Esther Smith, at 3 Grosvenor Place, many eulogistic speeches were made as to the methods of its work, and Mr. Asquith, K.C., M.P., moved a resolution "That it is necessary for the satisfactory medical attendance of the working classes in London that provident dispensaries, worked in co-operation with the hospitals, under rules generally approved by the medical profession, should be formed in every district of the metropolis on the system of the Metropolitan Provident Medical Association." In moving another resolution, Mr. Bousfield stated that the association adopted the principle of a wage limit. If these resolutions are to be taken as fairly describing the position occupied by this association, we can have no doubt that it will receive the hearty co-operation of the medical profession, and if it has in any degree failed to receive that co-operation we can only suppose that it has failed to convince the profession on the subject.

We are told that the membership amounts to more than 30,000 persons, whose payments in 1900 amounted to about £6,000. If, then, we take these figures as approximately expressing the status of the subscribing members, we find that they pay about four shillings a year each, and if all this goes to the medical men we do not think that they have much to complain of, on the understanding, of course, that the affair is a charity, that there is a wage limit, and that the dispensaries are so arranged as not to cut into private practice. We fancy, however, that there are many deductions before the money reaches the doctors' pockets, while we are sure that the subscribing members by no means regard themselves as the recipients of charity; and we should like to know somewhat more definitely what is the form which the "co-operation with the hospitals" takes, and what are the arrangements for enforcing a wage limit, especially in those dispensaries which have already been established and are now worked by independent committees. As things stand at the present time the dispensaries established by and connected with the Metropolitan Provident Medical Association are probably among the most respectable in London, and so long as this association remains under the guidance of those who have hitherto held the leading strings we have every confidence in its management. We confess, however, that we see but little guarantee as to the continued good conduct of such dispensaries and local committees as chose to drift into evil courses.

The difficulty arises when they cease to be charities. Right as it may be for medical men to practice under lay committees in the conduct of great charities, such as hospitals, it is not right for them to allow their professional services to be farmed out by lay committees as a matter of business, and in proportion as the relation between the doctor and the patient of a provident dispensary becomes a matter of business rather than of charity—which we know many of the subscribers to these dispensaries hold to be the case already—so should all middlemen be eliminated, and the whole control of the affair should be in the hands of the doctors.

There can be no doubt of the existence of a large class of people who can only pay for medical attendance by means of provident dispensaries; but if the medical men of any district wish to restrict the benefits of the penny-a-week dispensary to the proper penny-a-week class, they must take the trouble to establish these dispensaries themselves, and keep the whole management in their own hands. They are, however, too indifferent, perhaps too doubtful about each other's motives and good faith, perhaps too distrustful of their own business capacity; and so they prefer to stand aside and grumble, allowing commercial speculators, such as the various medical aid associations, or philanthropists such as the Medical Provident Association, to do what they ought to do for themselves. Fortunate is it for them when it is the philanthropists who take the field. But the whole position is wrong.

